# A Comparison of Cardiovascular Biomarkers in Patients Treated for Three Months with Etoricoxib, Celecoxib, Ibuprofen, and Placebo

**DOI:** 10.1111/j.1753-5174.2007.00002.x

**Published:** 2008-07

**Authors:** Christopher P Cannon, Cong Chen, Sean P Curtis, John Viscusi, Tuli Ahmed, Peter M DiBattiste

**Affiliations:** * TIMI Study Group, Cardiovascular Division, Brigham and Women's HospitalBoston, MA, USA; † Merck Research LaboratoriesWest Point, PA, USA; ‡ Merck Research LaboratoriesRahway, NJ, USA

**Keywords:** Etoricoxib, CRP, LDL-C, Homocysteine, Fibrinogen, Cardiovascular

## Abstract

**Objectives:**

Selective cyclooxygenase (COX)-2 inhibitors are effective analgesic and anti-inflammatory agents with improved gastrointestinal safety and tolerability compared with traditional NSAIDs. However, data from long-term, placebo-controlled studies have shown an increased risk of thrombotic cardiovascular (CV) events for COX-2 inhibitors. Changes in levels of CV biomarkers are potentially useful surrogate measures of pathologic changes associated with CV risk.

**Methods:**

We randomized 433 patients with osteoarthritis to etoricoxib 90 mg once daily, celecoxib 200 mg twice daily, ibuprofen 800 mg three times daily, or placebo for 12 weeks. The hypothesis was that etoricoxib would be non-inferior or superior to placebo in effect on C-reactive protein (CRP), LDL-cholesterol, homocysteine, and fibrinogen.

**Results:**

Relative to placebo, etoricoxib was noninferior for effect on CRP (decreased 7.8% vs. placebo; 97.5% CI of the difference: −30.5, 22.4), LDL-C (−4.0% vs. placebo; 97.5% CI: −10.6, 3.2), homocysteine (−3.9% vs. placebo; 97.5% CI: −11.6, 4.6), and fibrinogen (−3.7% vs. placebo; 97.5% CI: −9.4, 2.3). Etoricoxib was not different from placebo, celecoxib, or ibuprofen for any biomarker.

**Conclusion:**

Etoricoxib was comparable to placebo, celecoxib, and ibuprofen for effects on the CV risk markers measured.

## Introduction

Recent data suggest that long-term use of both traditional NSAIDs and cyclooxygenase-2 (COX-2) selective NSAIDs may increase cardiovascular (CV) risk compared with placebo [1–4]. Although several mechanistic hypotheses have been explored [5], the reason(s) for this increased risk still remains unclear and the subject of further investigation.

Etoricoxib is a selective COX-2 inhibitor used for the treatment of pain and inflammation, and is associated with a reduced risk of gastrointestinal clinical events and intolerability compared to traditional NSAIDs [6–9]. The recently completed multinational etoricoxib and diclofenac arthritis long-term (MEDAL) Program provided a direct and formal non-inferiority comparison of CV risk with the most widely used traditional NSAID diclofenac [10]. In the MEDAL Program, the risk of CV events associated with chronic, long-term administration of etoricoxib 60 or 90 mg daily, and diclofenac 150 mg per day in osteoarthritis and rheumatoid arthritis patients were shown not to be different from one another in arthritis patients with a variety of baseline risk factors [11].

We chose to examine the effects of etoricoxib and comparator NSAIDs on four well-characterized biomarkers of cardiac risk. Low-density-lipoprotein cholesterol (LDL-C) [12–14] is a well established marker of cardiac risk, and homocysteine [15,16], fibrinogen [17–19], and C-reactive protein [20–23] (CRP) are considered potential markers [24,25]. These biomarkers represent different processes implicated in the genesis of clinical CV disease—atherosclerosis, thrombosis, and inflammation. Elevation in the levels of each of these has been shown to be associated with CV risk [24,25]. In this study, etoricoxib, the COX-2 selective inhibitor celecoxib, and traditional NSAID ibuprofen were tested at their maximum recommended chronic doses in patients with osteoarthritis. Etoricoxib 90 mg/day is recommended for chronic symptomatic treatment of rheumatoid arthritis. Celecoxib 400 mg/day is the approved dosage for osteoarthritis and the recommended dosage for rheumatoid arthritis. Ibuprofen 2,400 mg/day is within the range of the recommended daily dose for osteoarthritis (1,200–3,200 mg/day).

## Methods

Etoricoxib protocol 065 was a 12-week, randomized, parallel-group, double-blind, placebo-controlled trial performed at 60 centers in the United States (see Appendix for list of investigators). The study group consisted of men and women aged ≥40 years old who had been diagnosed with osteoarthritis and treated with NSAIDS and/or COX-2 inhibitors within the previous year. Patients were instructed not to take non-study NSAIDs/COX-2 inhibitors and aspirin during the study. Exclusion criteria included inflammatory arthritis or any other systemic inflammatory disease, gastrointestinal malabsorption, evidence of significantly impaired renal function (creatinine clearance <30 mL/min), moderate to severe congestive heart failure (NYHA Class III or IV), history of atherosclerotic cardiac disease, uncontrolled hypertension, history of cerebrovascular disease, a bleeding diathesis, and active hepatic disease. Patients were also excluded if they were taking oral contraceptives or hormone replacement therapy, or were pregnant or nursing. Women of childbearing potential had to have a negative serum β-hCG level at screening and were instructed to use contraception measures. All participants provided written informed consent. The study was approved by a centralized institutional review board for the majority of the study sites and by the specific review board for the institution at the remaining sites. The study was conducted according to the principles of the Declaration of Helsinki.

Patients who had taken an NSAID or COX-2 inhibitor any time within the 2 weeks prior to screening (visit 1) underwent a 14-day washout, returned for evaluations (visit 2), and returned after one week to be randomized to treatment (visit 3, baseline). All other patients returned one week after screening to be randomized to treatment. After clinical exams were complete, all eligible patients were randomized according to a computer-generated, sponsor-supplied random allocation schedule in a 1:1:1:1 ratio to one of the following four treatments: etoricoxib 90 mg once daily, celecoxib 200 mg twice daily, ibuprofen 800 mg three times daily, or placebo. In addition, they received matching placebos for the other three treatments. Patients were assigned an allocation number in consecutive, ascending order from the block of allocations that each specific site received. Blinding was maintained using bottles labeled with the patients' allocation number. Compliance was determined from a pill count at each study visit. If more than 20% of the doses for an individual bottle was missed between two consecutive visits, the patient was considered noncompliant.

Patients had fasting blood sampling at screening, randomization (baseline) and 6 and 12 weeks (3 months). Biomarkers were analyzed by PPD Global Central Labs (Highland Heights, Kentucky). This laboratory maintains continuous certification by the CDC lipids program to ensure assay accuracy, and participates in the CAP Proficiency program for CRP, fibrinogen, and homocysteine. CRP was measured in serum or plasma using a high-sensitivity automated assay on the Behring Nephelometer II and mouse monoclonal CRP antibodies coated on polystyrene particles. The assay was linear in the range 0.04–2,500 mg/L. For the measurement of LDL-C (beta-quant), plasma was subjected to ultracentrifugation, and the fractions were analyzed on a Hitachi 747 Chemistry analyzer. The range of linearity for LDL-C was 3–1,000 mg/dL. Homocysteine was quantitated in plasma or serum via isocratic high-pressure liquid chromatography. The internal standard was L-homocystine, a dimer form of L-homocysteine, and assay sensitivity was in the range 1.0–80 µM. Plasma fibrinogen was measured via immunoassay on a Behring Nephelometer using rabbit anti-human antisera. The range of linearity was 60–976 mg/dL. Each biomarker assay was run with low and high quality control samples. The precision (percent coefficient of variation) of the quality control samples was in the range of 3.9–4.6% for the CRP assay, 3.1–3.3% for LDL-C (beta quant), 3.6–5.8% for homocysteine, and 3.0–4.6% for fibrinogen.

Physical exam, electrocardiogram, and complete fasting laboratory tests were performed during screening. Vital signs and body weight were evaluated at screening, randomization, week 6, and week 12. Adverse events were collected throughout the study and monitored from screening through 14 days after the final dose of study drug. At each visit, patients were questioned as to whether any adverse experience had occurred since the prior visit. Investigators evaluated adverse experiences with regard to severity (i.e., mild, moderate, or severe) and the likelihood of a relation to study medication. All adverse experiences were reported to the study sponsor and adverse experiences deemed to be serious (i.e., resulted in death, prolonged an existing hospitalization, lead to disability, or caused a birth defect in offspring of patients) were reported within 24 hours. All potential thrombotic CV events and deaths, regardless of cause, were adjudicated by an independent expert Case Review Committee (see Appendix) that was blinded to treatment assignment, as were upper gastrointestinal events (perforations, ulcers, bleeds) according to previously described criteria [26].

The clinical supplies were packaged according to the allocation schedule and blinded to the investigator, patient, and sponsor representatives. The official clinical database was not unblinded until medical and scientific review had been completed, protocol violators had been identified, per-protocol and modified-intention-to-treat populations had been defined, and the database had been declared complete.

### Data Analysis

The primary statistical analysis used the per-protocol population, according to clinical trial guidelines [27–29]. The per-protocol population consisted of patients who had valid data at randomization, had at least one post-randomization visit, did not take prohibited medication, had ≥80% compliance with study drug, and had their final biomarker sample drawn within the established window for month 3 (day 84 ± 14 days). Supportive analyses were based on the modified-intention-to-treat population, which consisted of patients with valid data at randomization and valid data from the month 3 visit (or week 6 visit for the week 6 analysis), regardless of whether they deviated from the protocol. Analyses of clinical and laboratory adverse experiences used data from all randomized patients.

Two primary hypotheses were tested hierarchically: etoricoxib 90 mg once daily would be (i) non-inferior to placebo in its effect on mean change from baseline for each biomarker after 3 months of treatment; and (ii) non-inferior to celecoxib 200 mg twice daily in its effect on mean change from baseline for each biomarker after 3 months of treatment. To maintain a 5% type I error rate for the overall hypothesis, each comparison (i.e., etoricoxib vs. placebo and etoricoxib vs. celecoxib) was conducted at the 2.5% level. Therefore, 97.5% CIs were provided. For etoricoxib to be declared non-inferior to placebo, the upper bound of the 97.5% CI for the relative difference in percentage points had to satisfy the following joint non-inferiority criteria: <20% for LDL-C, fibrinogen, and homocysteine; and <80% for CRP. These margins were based on variability in the markers, as observed in prospective epidemiological studies [30–32]. From these epidemiological data, the standard deviations for LDL, homocysteine and fibrinogen after log-transformation were comparable at ∼0.28; it is greater for CRP at 0.98. The noninferiority bounds were chosen to make sure that we would have comparable power for each biomarker. They each also roughly corresponded to a cardiovascular risk difference at ∼25%. If etoricoxib were found to be non-inferior to placebo, then further testing was to be conducted to determine superiority to placebo, and only non-inferiority testing (and no superiority testing) was to be conducted for the comparison between etoricoxib and celecoxib. If etoricoxib did not meet non-inferiority criteria compared to placebo, then etoricoxib would be further compared with celecoxib using the same criteria. If etoricoxib was non-inferior to celecoxib, then the second primary hypothesis was considered to have been met. A secondary hypothesis was that etoricoxib would be non-inferior to ibuprofen: for etoricoxib to be declared non-inferior, the upper bounds of the four (2-sided) 95% CIs for the relative difference had to meet the same non-inferiority margins as for the primary hypothesis (<20% for LDL-C, fibrinogen, and homocysteine; and 80% for CRP). The overall type I error rate was 5% (two-sided) for the primary hypothesis. This was achieved by adjusting the type I error rate for each of the two primary comparisons to 2.5% (two-sided). Within each comparison, all biomarkers must have met the prespecified criteria. With this conservative approach, which can only decrease the significance level, no further multiplicity adjustment was required. For superiority testing, the overall type I error rate was 5% (two-sided). Because the superiority hypothesis was nested within the non-inferiority hypothesis, no extra multiplicity adjustment was needed for the duality of the testing.

The analyses used standard longitudinal data analysis techniques suited for repeated measures. For each biomarker, log-transformed levels were fit to a mixed-effects model. The mean difference in change from baseline (randomization) at month 3 between etoricoxib and the comparator (etoricoxib minus comparator), along with a per-protocol, multiplicity-adjusted, two-sided 97.5% CI were calculated using the log-transformed data. They were back-transformed exponentially to obtain their corresponding relative differences and 97.5% CIs in the raw scale.

The study was powered to enroll enough patients to have 75 patients per group in the per-protocol population, such that the probabilities to meet the individual non-inferiority criteria were 96% for LDL-C, 96% for homocysteine, 97% for fibrinogen, and 93% for CRP. Assuming the four biomarkers are independent of each other, the overall power to demonstrate non-inferiority of etoricoxib to placebo on all four markers was conservatively estimated to be 83%. The power analysis was performed before the study and was pre-specified in the study protocol. No interim analyses were conducted. Analysis of clinical adverse experiences and clinical safety were based on all patients randomized.

Prespecified subgroup analyses were conducted according to gender, age (> or ≤median), history of diabetes, history of hypertension, current tobacco use, and 10-year Framingham (LDL-based) coronary heart disease risk score [33] (> or ≤median) to further explore the comparison between etoricoxib and placebo.

A pre-specified exploratory analysis was conducted to project the relative risk of a major adverse CV event between etoricoxib and each comparator at month 3 for the per-protocol population. The analysis was conducted using the levels of the individual biomarkers and the associated cardiac risk reported in the literature [30–32,34], along with the observed correlations among the biomarkers from this study. To account for the projected impact of all four markers on the cumulative risk in each treatment group, a formula was developed from the incremental risks associated with incrementally higher levels of the biomarker, as reported in the literature. The basic statistical idea is illustrated in the following example. Consider two correlated biomarkers with a correlation of θ. Suppose that historical data show that cardiac risk increases 1% if the level of either biomarker increases by one unit. Then, by the statistical methods used in this study, the projected cardiac risk would increase by 2/(1 + θ)% if the levels of both biomarkers simultaneously increase by one unit.

## Results

**Figure 1:**
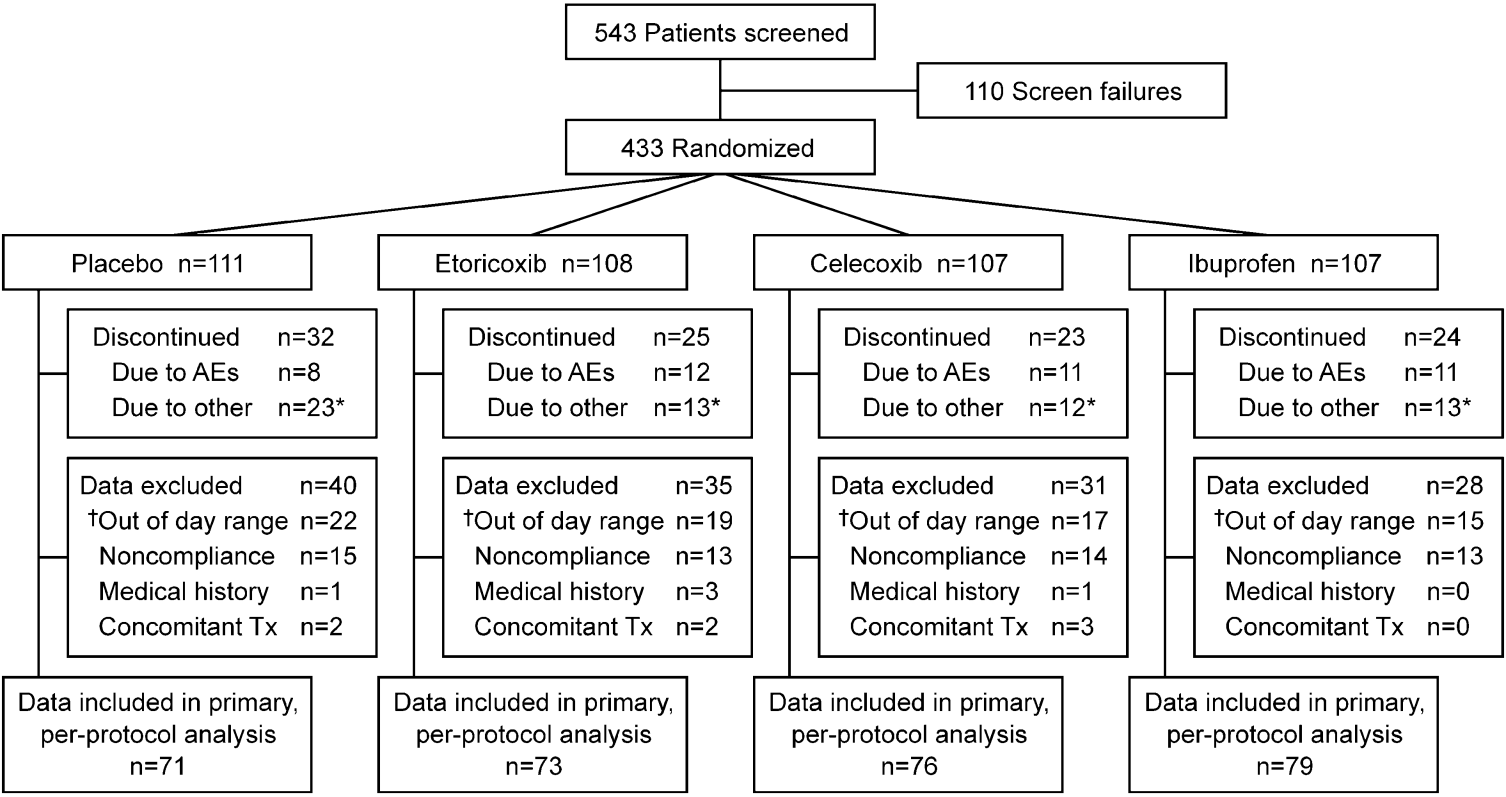
Patient accounting. *Includes lack of efficacy, protocol deviation, lost to follow-up, moved, withdrew consent, and miscellaneous reasons. †Data were out of day range because data were collected outside of the protocol defined window (±14 days) for pre-specified biomarker analysis time points.

The study was conducted from September 30, 2002 to July 4, 2003. A total of 54 sites randomized 433 patients, 299 of whom met the criteria for the per-protocol analysis (Figure 1). Reasons for study drug discontinuation were similar across the four treatment groups.

Baseline demographics and vital signs were similar across the four treatment groups (Table 1). Additionally, baseline demographics for patients included in the primary per-protocol analysis were similar to baseline demographics for all patients randomized in the study. Arithmetic mean CRP values ranged from 4.6 mg/L to 6.0 mg/L at randomization, with the etoricoxib group having the lowest mean value and the celecoxib group having the highest. CV biomarker values at randomization were otherwise similar across treatment groups (Table 2). For patients who underwent a washout, comparison of biomarker values between visits 2 and 3 showed that all biomarkers appeared to have achieved a steady state, suggesting that any effect of prior treatment with NSAIDs or COX-2 inhibitors on these markers had dissipated during the washout period.

**Table 1:** Baseline patient characteristics

	Placebo (N = 111)	Etoricoxib (N = 108)	Celecoxib (N = 107)	Ibuprofen (N = 107)
Mean age (SD) (years)	57.8 (10.1)	59.3 (10.6)	59.3 (10.3)	56.8 (10.2)
Sex [n, (%)]				
Women	75 (67.6)	68 (63.0)	64 (59.8)	62 (57.9)
Current tobacco user [n, (%)]	19 (17.1)	14 (13.0)	11 (10.3)	18 (16.8)
History of [n, (%)]				
Diabetes	6 (5.4)	4 (3.7)	4 (3.7)	3 (2.8)
Hypertension	48 (43.2)	48 (44.4)	39 (36.4)	43 (40.2)
Hypercholesterolemia	20 (18.0)	19 (17.6)	18 (16.8)	20 (18.7)
Framingham Risk Score >Median (6) [n, (%)]	39 (35.1)	42 (38.9)	44 (41.1)	41 (38.3)

**Table 2:** Change in biomarker measurements and select vital signs from randomization to week 12 for patients in the per-protocol population

	Placebo		Etoricoxib		Celecoxib		Ibuprofen	
	N	Mean (SD)	N	Mean (SD)	N	Mean (SD)	N	Mean (SD)
Measurements of Biomarkers Throughout the Study Duration								
CRP (mg/L)								
Randomization	111	5.4 (6.0)	108	4.6 (6.1)	107	6.0 (6.9)	106	5.2 (5.8)
Week 12	70	5.2 (6.1)	72	4.0 (4.6)	76	4.6 (5.3)	79	4.4 (5.4)
LDL-C (mg/dL)								
Randomization	110	127.2 (37.6)	108	121.2 (28.7)	107	131.1 (34.8)	105	124.8 (30.9)
Week 12	69	123.2 (29.5)	72	115.5 (29.2)	76	127.7 (34.4)	79	124.2 (30.9)
Homocysteine (µmol/L)								
Randomization	111	9.0 (3.0)	107	8.9 (3.0)	107	9.6 (9.1)	107	9.2 (2.6)
Week 12	71	8.9 (2.7)	72	8.8 (3.2)	76	8.6 (2.4)	79	9.3 (2.9)
Fibrinogen (mg/dL)								
Randomization	110	418.5 (81.7)	107	409.8 (84.5)	107	420.6 (86.7)	106	410.3 (82.7)
Week 12	71	413.0 (85.3)	73	388.1 (87.8)	76	396.6 (86.1)	78	386.6 (74.8)
Measurements of Blood Pressure and Body Weight Throughout the Study Duration								
Systolic Blood Pressure								
Randomization	67	125.5 (31.1)	69	126.7 (14.2)	63	128.3 (13.0)	73	127.1 (14.4)
Week 12	67	126.7 (14.4)	69	130 (14.9)	63	131.8 (14.4)	73	127.8 (16.2)
Diastolic Blood Pressure								
Randomization	67	78.3 (7.7)	69	77.2 (7.1)	63	77.3 (7.0)	73	79.2 (8.0)
Week 12	67	78.9 (9.0)	69	78.3 (9.2)	63	78.3 (7.9)	73	77.1 (8.2)
Weight (lb)								
Randomization	67	186.3 (43.2)	69	188.9 (47.1)	63	205.5 (44.8)	71	186.2 (36.4)
Week 12	67	186.6 (43.0)	69	190.8 (47.6)	63	205.4 (44.3)	71	188.9 (37.2)

**Figure 2:**
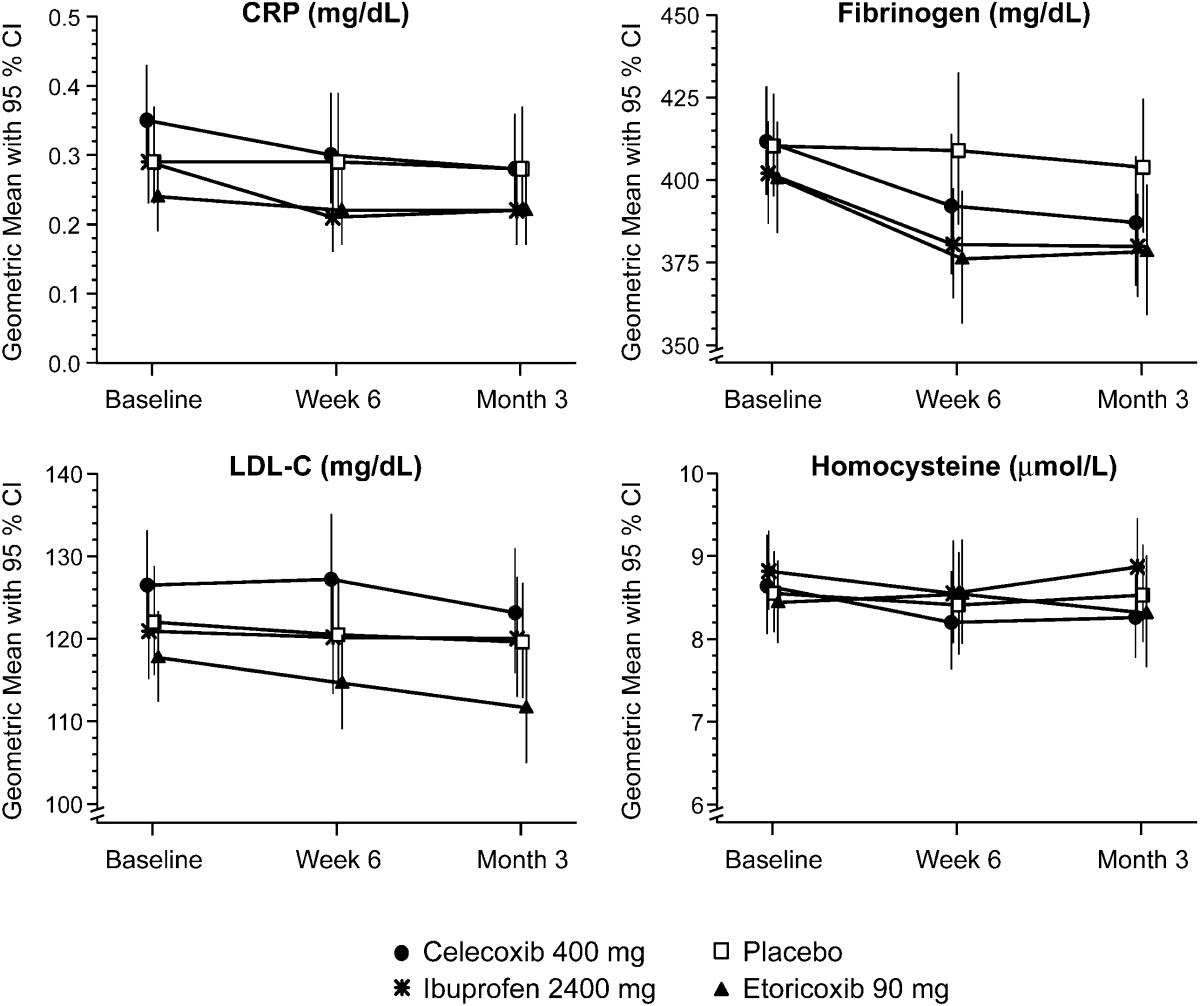
Geometric mean biomarker measurements across treatment groups in the per-protocol population.

Figure 2 shows the geometric mean values of the biomarkers at randomization, week 6, and month 3 across the treatment groups. In the ibuprofen group, the level of homocysteine increased, and the other biomarkers decreased. Table 2 shows mean biomarker measurements at randomization and at month 3; Table 3 shows the results of the between-group comparisons of mean change. At month 3, etoricoxib was non-inferior to placebo in its effect on the 4 biomarkers in the per-protocol analysis (Table 3), satisfying the primary hypothesis. The upper bounds of the CIs for the relative differences for the comparisons between etoricoxib and placebo fell well below the prespecified non-inferiority margin of 80% for CRP and below 20% for fibrinogen, LDL-C, and homocysteine. The effects of etoricoxib on all four biomarkers were non-inferior to those observed for both celecoxib and ibuprofen (Table 3). Analyses of the modified-intention-to-treat population showed similar results (data not shown). In the per-protocol population, the month 3 results were generally in agreement with the week 6 results, except that for the etoricoxib-vs-placebo comparison, the relative difference (in raw scale) between the groups for CRP was greater at week 6 than at month 3 (−18.1% at week 6 vs. −7.8% at month 3), and for the etoricoxib-vs-celecoxib comparison, the relative difference in CRP was smaller at week 6+ than at month 3 (1.8% at week 6 vs. 23.1% at month 3).

**Table 3:** Difference between etoricoxib and comparators in change in biomarker concentrations from randomization at month 3

	Relative difference (Etoricoxib minus Comparator) [% (CI)†]	Upper bound of CI meets non-inferiority criterion?‡	*P*-Value Superiority testing of etoricoxib vs. comparator§,¶
	Comparator		
	Placebo		
CRP	−7.8 (−30.5, 22.4)	Yes	0.52
LDL-C	−4.0 (−10.6, 3.2)	Yes	0.20
Homocysteine	−3.9 (−11.6, 4.6)	Yes	0.30
Fibrinogen	−3.7 (−9.4, 2.3)	Yes	0.16
	Celecoxib		
CRP	23.1 (−6.8, 62.7)	Yes	—
LDL-C	−1.4 (−7.4, 4.9)	Yes	—
Homocysteine	−0.4 (−8.9, 9.0)	Yes	—
Fibrinogen	2.5 (−3.6, 9.0)	Yes	—
	Ibuprofen		
CRP	14.0 (−10.1, 44.6)	Yes	—
LDL-C	−2.9 (−8.0, 2.6)	Yes	—
Homocysteine	−3.0 (−10.5, 5.2)	Yes	—
Fibrinogen	1.0 (−3.9, 6.3)	Yes	—

† CI was 97.5% for etoricoxib vs. placebo and etoricoxib vs. celecoxib, and 95% for etoricoxib vs. ibuprofen.

‡ Test whether upper bound of CI for relative difference is less than non-inferiority margin (i.e., <80% for CRP and <20% for LCL-C, homocysteine, and fibrinogen).

§ Applicable only for the etoricoxib-vs-placebo comparison and only if etoricoxib was shown to be non-inferior to placebo.

¶ Longitudinal analysis with repeated measures in a per-protocol population. Mixed model included factors for treatment, visit, treatment-by-visit interaction, gender, current tobacco use, diabetes, hypertension, age category (> or ≤median), and Framingham risk score as fixed effects using an unstructured covariance matrix.

### Subgroup Analyses

Subgroup exploratory analyses of the etoricoxib-vs-placebo comparison in the per-protocol population at month 3 showed results generally consistent with the primary analysis. The numbers of diabetic patients or current tobacco users were small in each group. With the caveat that the large number of tests could yield some significant results by chance alone, the results showed that certain biomarkers decreased significantly after etoricoxib treatment compared with placebo in some subgroups. In particular, among current tobacco users, CRP in the etoricoxib group decreased significantly below placebo (−46.3% vs. placebo, 95% CI of the relative difference between groups: −65.2%, −17.2%). Also in the etoricoxib group, CRP decreased 50.8% (95% CI: −81.7, 32.5) below placebo among diabetic patients and 31.9% (95% CI: −55.0, 3.1) below placebo in individuals with higher than median Framingham risk scores; while these treatment differences were noteworthy, they did not reach statistical significance. No biomarkers increased significantly after etoricoxib compared with placebo in the subgroup analyses.

### Tolerability

The percentages of patients with at least one clinical adverse experience, with clinical adverse experiences deemed by the investigator as being possibly related to study drug, and who discontinued due to such drug-related adverse experiences were lowest in the placebo group and generally similar across the active treatment groups (Table 4). The incidence of patients who discontinued due to any clinical adverse experience was similar across all groups.

**Table 4:** Clinical adverse experiences

	Placebo N = 111 n (%)	Etoricoxib 90 mg qd N = 108 n (%)	Celecoxib 400 mg qd N = 107 n (%)	Ibuprofen 2,400 mg qd N = 107 n (%)
Clinical Adverse Experiences (AEs)				
≥1 AE	40 (36.0)	50 (46.3)	45 (42.1)	45 (42.1)
≥1 Drug-related AE†	13 (11.7)	20 (18.5)	15 (14.0)	20 (18.7)
≥1 Serious AE	0 (0.0)	1 (0.9)	4 (3.7)	2 (1.9)
Discontinued due to an AE	8 (7.2)	12 (11.1)	11 (10.3)	11 (10.3)
Gastrointestinal (GI) Nuisance Symptoms‡				
GI Symptom AEs	2 (1.8)	7 (6.5)	4 (3.7)	6 (5.6)
Discontinuations	0 (0.0)	2 (1.9)	0 (0.0)	2 (1.9)
Renovascular AEs				
Lower Extremity Edema	0 (0.0)	0 (0.0)	0 (0.0)	0 (0.0)
Discontinuations	0 (0.0)	0 (0.0)	0 (0.0)	0 (0.0)
Congestive Heart Failure	0 (0.0)	0 (0.0)	0 (0.0)	0 (0.0)
Hypertension§	1 (0.9)	4 (3.7)	1 (0.9)	4 (3.7)
Discontinuations	0 (0.0)	2 (1.9)	0 (0.0)	0 (0.0)

† Determined by the investigator to be possibly, probably or definitely drug related.

‡ Includes abdominal pain, abdominal discomfort, acid reflux, dyspepsia, epigastric discomfort, heartburn, nausea and vomiting.

§ Hypertension was diagnosed by individual investigators; a formal set of parameters was not provided.

The three nonfatal potential investigator-reported thrombotic CV events that were reported in this trial were adjudicated by an external committee as part of an established adjudication process for all randomized clinical trials of etoricoxib of at least 4 weeks in duration. One of these events was confirmed, an acute myocardial infarction in a patient in the celecoxib group. The adjudication committee determined that there was not enough information for the other two events to confirm them as thrombotic CV events; these events included a traffic accident that lead to death (this patient was not randomized to study medication) and a neurological disorder in a patient receiving etoricoxib. There were no significant upper gastrointestinal clinical adverse experiences (perforations, ulcers, or upper gastrointestinal bleeding). With regard to prespecified adverse experiences of special interest (i.e., digestive and renovascular), rates of these adverse experiences and discontinuations due to these experiences were generally similar across groups (Table 3). There were no deaths among randomized patients in this study.

## Discussion

Both COX-2 selective inhibitors and traditional NSAIDs, with the exception of high-dose naproxen, have been associated with an increased risk of thrombotic CV events. The mechanism by which these therapeutic agents increase this risk is currently unknown although the inhibition of PGE_2_and prostacyclin, a prostanoid that effects mediators associated with platelet activation, hypertension, and atherogenesis, has been suggested as an explanation [35]. The current study was conducted in order to investigate whether other factors associated with increased CV risk could be related to the increased CV risk of these agents. We investigated the effect of etoricoxib on four biomarkers of CV risk, i.e., CRP, LDL-C, homocysteine, and fibrinogen, implicated in the genesis of clinical CV disease with roles in atherosclerosis, thrombosis, and inflammation [24].

Treatment with etoricoxib 90 mg for three months in patients with osteoarthritis was non-inferior to placebo, celecoxib, and ibuprofen with respect to its effects on all four biomarkers. Moreover, treatment with etoricoxib 90 mg for up to 3 months did not elevate the levels of any of the biomarkers studied. These findings, do not offer any alternative hypotheses for the increased risk of CV events compared to placebo observed with COX-2 selective inhibitors and traditional NSAIDs beyond those that have been most studied [35].

While each of the biomarkers evaluated in this study has been shown to predict CV risk, it is important to note that they should not be considered a substitute for clinical outcomes. The current study was not of sufficient duration or adequately powered to draw any conclusions about the effects of etoricoxib, celecoxib, or ibuprofen on CV clinical outcomes. Data from the MEDAL Program, a CV clinical outcomes program that evaluated etoricoxib and the traditional NSAID diclofenac, has recently become available [36]. Additionally, other outcomes programs that aim to evaluate CV clinical outcomes of other agents have been initiated [37].

In summary, the effects of etoricoxib on CRP, LDL-C, homocysteine, and fibrinogen were comparable with those of placebo, celecoxib, and ibuprofen in patients with osteoarthritis. While an absence of an adverse effect of etoricoxib on established CV biomarkers over 3 months cannot be interpreted as evidence of absence of a clinical CV risk, the data from the current study provide important information about the effects of selective COX-2 inhibition on these recognized markers of CV risk.

## Appendices

### Investigators

Lawrence K. Alwine, D.O., Dowingtown, PA; Allan B. Aven, M.D., Arlington Heights, IL; Herbert S.B. Baraf, M.D., Wheaton, MD; Thomas D. Bianchi, M.D., Tallassee, AL; Leo P. Bidula, M.D., Pittsburgh, PA; B. Ian Blatt, M.D., Havertown, PA; David G. Borenstein, M.D., Washington, DC; Robert Burton, M.D., Anaheim, CA; Teresa Coats, M.D., Austin, TX; Lydia G. Corn, M.D., Sarasota, FL; James E. Dowd, M.D., P.C., Howell, MI; John K. Earl, M.D., Hickory, NC; William M. Edwards, M.D., Charleston, SC; James I. Fidelholtz, M.D., Cincinnati, OH; Thomas Fiel, D.O., Tempe, AZ; Steven D. Folkerth, M.D., Las Vegas, NV; Neil Fraser, M.D., Troy, MI; David L. Fried, M.D., Warwick, RI; Robert R. Gao, M.D., Las Vegas, NV; Ronald M. Gilman, M.D., East Providence, RI; Geoffrey Gladstein, M.D., Stamford, CT; Ty H. Goletz, M.D., San Antonio, TX; Steven Goodman, M.D., Delray Beach, FL; Gary V. Gordon, M.D., Wynnewood, PA; Joseph S. Habros, M.D., Scottsdale, AZ; Shannon Howe, M.D., Tucson, AZ; Robert F. Hynd, M.D., Oklahoma City, OK; Terence Isakov, M.D., Lyndhurst, OH; Arvind Jariwala, M.D., Raleigh, NC; Beth L. Jonas, M.D., Chapel Hill, NC; Randolph P. Jones, M.D., Mission Viejo, CA; Roy A. Kaplan, M.D., Concord, CA; Rashid A. Khairi, M.D., Indianapolis, IN; Alan J. Kivitz, M.D., Duncansville, PA; Michael Kohen, M.D., South daytona, FL; Nelson P. Kopyt, D.O., Allentown, PA; Albert H. Lee, M.D., Norfolk, VA; Robert D. Madder, D.O., Beaver, PA; Clark D. McKeever, M.D., Houston, TX; John V. Murray, M.D., Pinellas Park, FL; Howard Offenberg, M.D., daytona Beach, FL; Andres Patron, D.O., Hollywood, FL; Edward Barry Portnoy, M.D., Westlake Village, CA; Adam Rosen, M.D., Largo, FL; Lawrence S. Schiffman, M.D., Northampton, MA; Gregory Serfer, D.O., Hollywood, FL; Stephan C. Sharp, M.D., Nashville, TN; Fredrica E. Smith, M.D., Los Alamos, NM; Timothy S. Truitt, M.D., Melbourne, FL; Michael K. Vandenberg, M.D., Pensacola, FL; Albert G. Volk, M.D., St. Augustine, FL; Alexander White, M.D., Orange City, FL; Michael S. Wukelic, M.D., Spokane, WA; Gary D. Yeoman, D.O., Philadelphia, PA; and Steven Zeig, M.D., Pembroke Pines, FL.

### Vascular Events Committee

George W. Vetrovec, M.D., Richmond, VA; Bernard R. Chaitman, M.D., St. Louis, MO; Leonard S. Dreifus, M.D., Heathrow, FL; Thom Rooke, M.D., Rochester, MN; Jeffrey Ginsberg, M.D., Hamilton, Ontario, Canada; Clive Kearon, Ph.D., Hamilton, Ontario, Canada; Justin A. Zivin, M.D., La Jolla, CA; Jay P. Mohr, M.D., New York, NY; and Harold P. Adams, M.D., Iowa City, IA.
